# Post-embryonic tail development through molting of the freshwater shrimp *Neocaridina denticulata*

**DOI:** 10.1016/j.isci.2025.111885

**Published:** 2025-01-23

**Authors:** Haruhiko Adachi, Nobuko Moritoki, Tomoko Shindo, Kazuharu Arakawa

**Affiliations:** 1 Institute for Advanced Biosciences, Keio University, Tsuruoka, Yamagata 997-0017, Japan; 2 Graduate School of Media and Governance, Keio University, Fujisawa, Kanagawa 252-0882, Japan; 3 Electron Microscope Laboratory, Keio University School of Medicine, Shinjuku, Tokyo 160-8582, Japan

**Keywords:** Biological sciences, Zoology, Developmental biology

## Abstract

*Neocaridina*, a crustacean, exhibits distinctive tail morphogenesis during molting, making it a potential model for post-embryonic morphogenesis. After the first molting, two significant changes occur in the tail: the uropod branches are cleared and the telson undergoes convergent elongation. Cross-sectional analysis showed that uropod and telson branching begins immediately after hatching as cuticle branching. The surface structure of the developing tail suggested that telson elongation is achieved through anisotropic cuticle furrow extension during molting, linked to epithelial cell shape changes occurring post-hatching. An *in vivo* live imaging system with UV-LED resin revealed gradual telson contraction before molting. Additionally, a draft genome of *Neocaridina denticulata* was provided. This research enhances understanding of arthropod morphogenesis through molting and lays groundwork for further developmental and cytological studies in *Neocaridina*.

## Highlights

• Uropod branching and telson convergent elongation occur during *Neocaridina*’s first molting
• Live imaging with UV-LED resin shows the cellular dynamic pre-molting tail changes
• The draft genome of *Neocaridina* has been sequenced

## Graphical abstract

**Figure:**
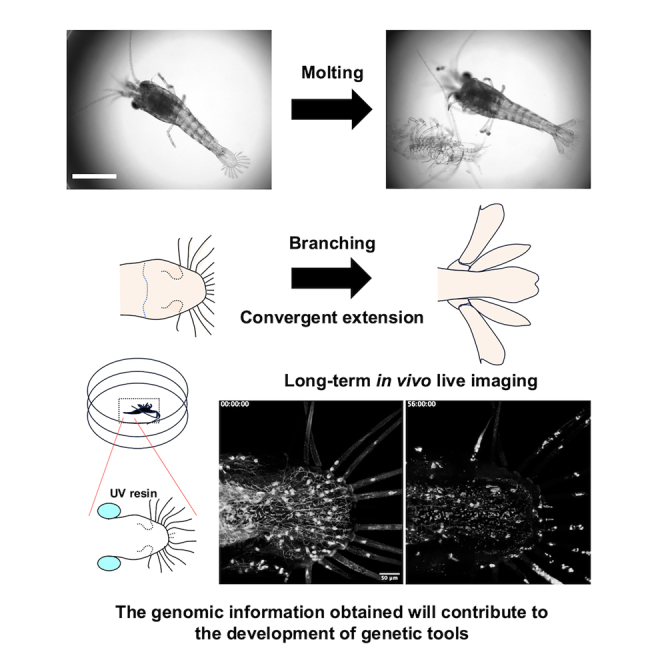

## Introduction

Understanding the morphogenesis of organisms is a crucial issue in developmental biology. Recently, attention has shifted toward not only embryogenesis but also post-embryonic morphogenesis, particularly in arthropods through molting.^1^ Among arthropods, insects are particularly well studied for this purpose. Insects can be classified into different modes of development, such as holometabolous, hemimetabolous, and ametabolous insects.^2^ The developmental process of metamorphosing holometabolous beetles and hemimetabolous treehoppers, respectively, involves universal hierarchical mechanisms. That is the unfolding of epithelial sheet folds and the formation of the folds of different scales.^1^^,^^3^^,^^4^^,^^5^^,^^6^ In particular, the Japanese beetle, *Trypoxylus dichotomus*, has been identified as a model organism for the study of three-dimensional horn formation during development, and there is the presence of a genetic basis for this process.^7^^,^^8^^,^^9^^,^^10^^,^^11^ In *Drosophila*, a model organism for holometabolous insects, the growth of imaginal discs has been extensively studied from molecular genetic to mechanistic perspectives.^12^^,^^13^ During the prepupal-pupal period, the individuals are macroscopically immobile while the morphogenetic process occurs. Therefore, *in vivo* live imaging studies are being performed in *Drosophila*.^14^^,^^15^^,^^16^ On the other hand, in hemimetabolous insects, particularly *Gryllus*, it is becoming possible to describe the developmental process and perform some advanced genetic manipulation,^17^^,^^18^ however, live imaging of morphological changes through molting is not yet available. This is because individuals move macroscopically until just before ecdysis, and are covered by a hard, colored cuticle. It is important to study how morphogenesis is achieved in individuals that remain in a state of macromotion until just before ecdysis. Recently, there has also been a growing focus on macro-movement and morphogenesis; it has been found that muscle-mediated macromotion plays a crucial role in morphogenesis in the cnidarian *Nematostella vectensis*.^19^ Among arthropods, crustaceans move macroscopically just before ecdysis, similar to hemimetabolous insects and some species in crustaceans also induce morphological changes through molting. Additionally, certain crustaceans may be suitable for the application of live imaging techniques, given their transparency. The utilization of live imaging techniques and the advancement of genetic tools will facilitate a comprehensive and detailed investigation of the underlying mechanisms of molting morphogenesis.

Genetic tools are currently being developed also for *Daphnia magna* as a research model for crustaceans.^20^^,^^21^^,^^22^ The marine amphipod *Parhyale* has also been recently used as a model organism for crustacean developmental and cellular phenomena.^23^ The whole genome sequencing has been achieved,^24^ and an amazing live imaging technique using adhesion has been established in *Parhylae*^25^ and *Parhylae* was used in the comparative analysis of leg regeneration and developmental phenomena.^26^^,^^27^ On the other hand, *Daphnia* and *Parhyale* have almost the same morphology as their parents from birth, with few tissues undergoing extreme morphogenesis through ecdysis. Therefore, we focused on the morphogenesis of *Neocaridina denticulata* (*Neocaridina davidi*)*.* A recent review has described *Neocaridina* as a potential model organism for decapods due to its ease of husbandry and reproduction.^28^ *Neocaridina* exhibits extreme changes of tail morphology through the first molt.^29^^,^^30^ Observational studies have been conducted on muscle and nerve development during the tail development using fixed specimens.^31^^,^^32^ However, the details of the tail morphogenesis through molting, including the short timescale dynamics, have not been fully clarified. Additionally, currently, there are no publicly available genome assemblies for *Neocaridina* that could facilitate the development of future genetic tools, although CRISPR/Cas9 knockout experiments with transcriptome data have recently been conducted in this species.^33^

In this study, we investigated tail development in *N. denticulata* through histological analysis and *in vivo* live imaging using fluorescent probes. Additionally, draft genomes were obtained using nanopore long-read sequencing to establish future genetic tools. This study contributes to a comprehensive understanding of the mechanisms of morphogenesis through molting in arthropods and future developmental and cytological studies in *Neocaridina*.

## Results and discussion

### The changes of the tail shape through first ecdysis

**Figure 1:**
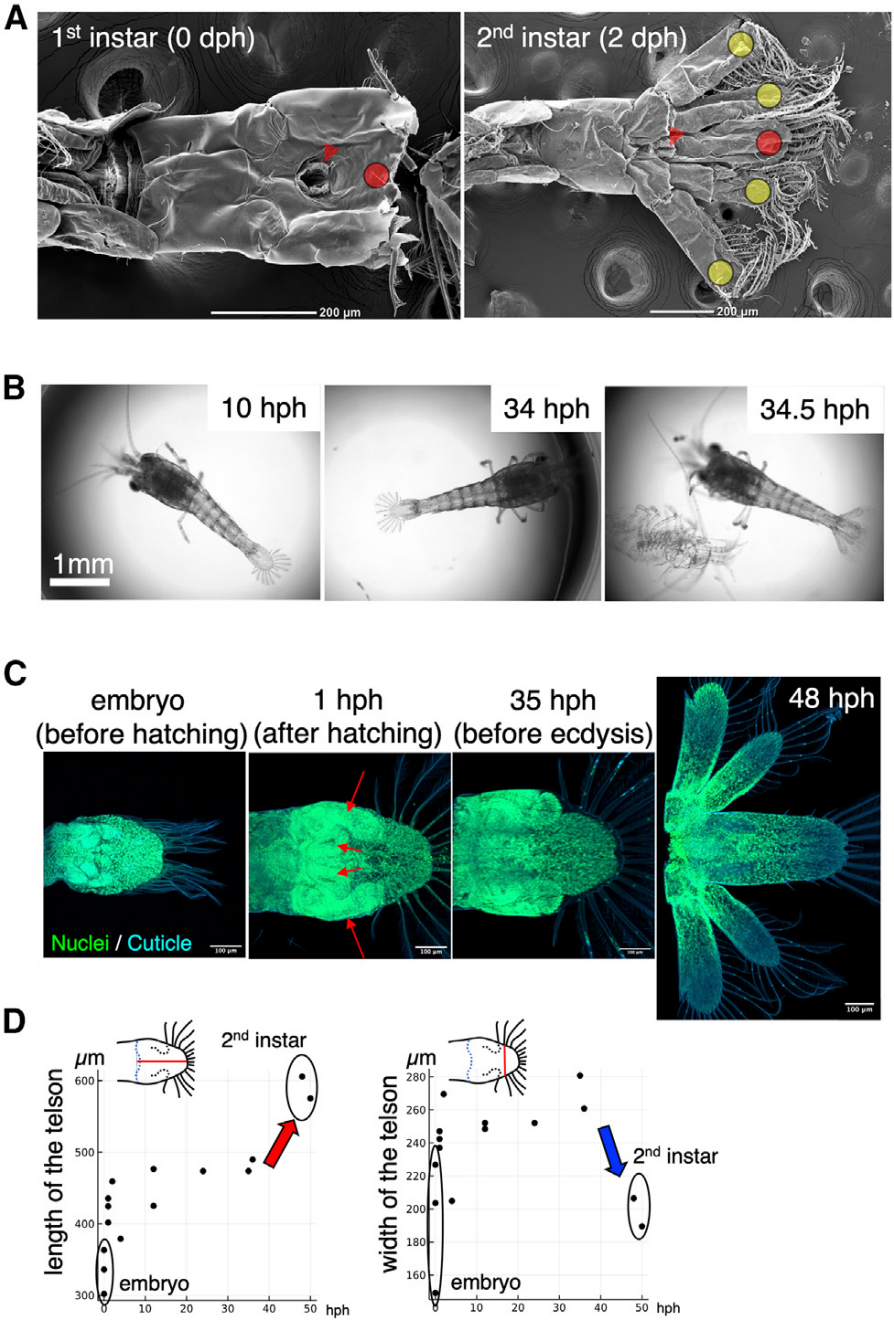
Morphogenesis of the tail of *Neocaridina* (A) SEM image of the tail of *Neocaridina*, 1st instar (0 days post-hatch) and 2nd instar (2 days post-hatch), taken from the ventral side. The red arrowhead indicates the anus. The yellow circle indicates the branched uropod and the red one indicates the telson. (B) Time-series images of molting. Extracts from the videos at 10 h post-hatching (10 hph), 34 h post-hatching (34 hph), and 34.5 h post-hatching (34.5 hph) are included. (C) The developmental tail was imaged using fluorescence. Nuclei were stained with SYTO13 and the cuticle was detected through autofluorescence upon exposure to a 405 nm wavelength laser. (D) Morphological changes over time were analyzed. The length of the specimen was measured from the root of the uropod to the tip of the telson, while the width was measured between the second setae on the proximal side. (*n* = 15 animals).

The structure of a shrimp tail comprises a telson and four uropods. *Neocaridina* undergoes significant changes in the uropod during the first ecdysis after hatching.^29^^,^^30^ Also in this study, scanning electron microscope (SEM) analysis confirmed that the branching structure corresponding to the uropods was not observed immediately after hatching, and that the branch appeared after ecdysis (Figure 1A). Furthermore, we recorded the ecdysis process in time-lapse and observed that the change in tail shape occurs within a few minutes (Figure 1B and Videos S1 and S2). The time-lapse imaging also revealed the development of the tail primordium with some level of detail. Although the presence of uropod primordia was unclear in the early stage, the branching of the primordia of uropods and telson became evident in the late stage (Figure 1B, Videos S1 and S2). In a millipede, *Nipponia nodulosa*, appendages also develop through molting, and it has been reported that they develop inside the transparent protrusion on the surface of the body segments.^34^ No such structure protruding was observed in the case of *Neocaridina* (Figure 1A). This could be due to structural differences between regular legs and uropods.

Video S1. Early stage of 1st instar development Neocaridina in 96 well plate Same sample as Video S2.

Video S2. Late stage and ecdysis of 1st instar Neocaridina in 96 well plate Same sample as Video S1.

Video S3. z stack imaging of phalloidin staining of 15 hph It is played back from the dorsal side to the ventral side.

Video S4. z stack imaging of phalloidin staining of 30 hph It is played back from the dorsal side to the ventral side.

Changes in tail shape during development were investigated using confocal microscopy. Nuclear staining and autofluorescence of the cuticle upon exposure to a 405 nm wavelength laser were used for visualization. The tail of the embryonic period was examined in addition to the time series since hatching. At around one week after incubation, the embryo begins to form an eye and make a beat. The tail was taken out by dissecting the embryo of that period. Upon hatching, bifurcated structures, namely the uropod primordia and the telson primordia, were observed (Figure 1C). The size of the telsons and telson primordia remained relatively constant between hatching and ecdysis, with notable changes occurring from embryo to hatching and through the first ecdysis. From the embryo stage to hatching, both the length and the width of the telson increased (Figure 1D). On the other hand, through the first ecdysis, the length increased while the width decreased (Figure 1D).

The initial molt results in two significant alterations to the tail. First, the branches of the uropods are cleared, and second, the telson undergoes convergent elongation.

### Time-series changes in the cross-sectional structure of the tail

**Figure 2:**
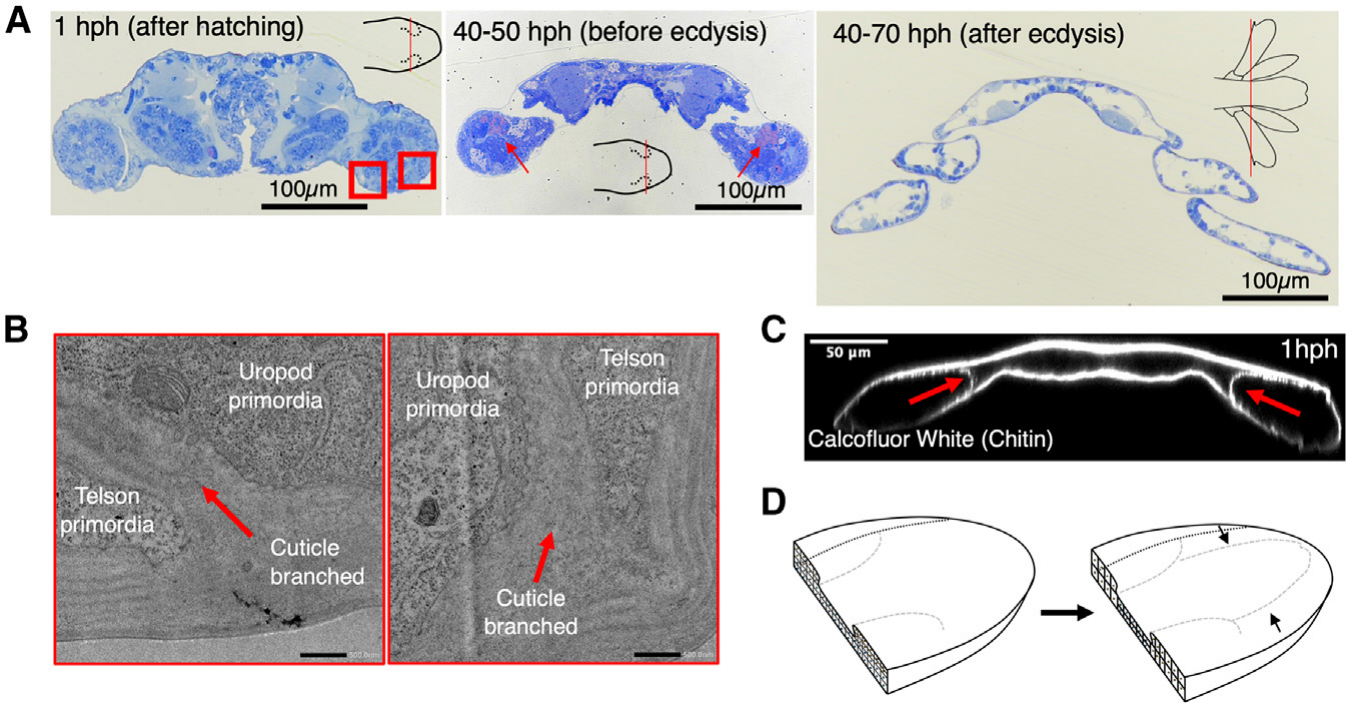
Cross-sectional structure of the developmental tail (A) Toluidine blue staining of semi-thin section. The plane of section in 1st instar larvae is exactly where the uropod primordium is present. The red rectangles indicate the enlarged areas in (B). The red arrows show toluidine blue-red-purple positive sites, which may indicate the presence of mucopolysaccharides. (B) Transmission electron microscopy (TEM) image from (A) red rectangle regions in 1 hph. The red arrows indicate the cuticular branch separating the uropod and telson primordia. (C) Optical section of calcofluor white staining tail in 1 hph. The red arrows show the cuticular branch separating the uropod and telson primordia. (D) Scheme of the cellular structure of the tail primordium during the molting process. Already in the early stages of development, the uropod and the telson primordium are present in an overlapping state (left), although separated by the cuticle, and the overlap is resolved during development (right).

The following analysis focused on changes in the cross-sectional structure of the tail. Toluidine blue staining was used to examine the cross-section of the tail during the development process (Figure 2A). The results showed that just before ecdysis, the uropod primordia and the telson primordia were completely separated. Shortly before ecdysis, the interstitial ECM structure of the toluidine blue-red hyaline was revealed (Figure 2A). It is believed that the structure may play a crucial role in causing morphogenesis through the first ecdysis within a few minutes, as it is not present immediately after hatching (Figure 2A). Further research is required to identify this molecule in the future. To investigate the boundary between the uropod primordia and the telson primordia immediately after hatching, we analyzed the microstructure using transmission electron microscopy (TEM). Immediately after hatching, the uropod primordia and telson primordia were found to be isolated by the cuticle (Figure 2B). In other words, the exoskeleton’s cuticle is already branched immediately after hatching. To confirm this, calcofluor white staining was performed, which stains chitin and cellulose. As a result, consistent with the results of TEM analysis, it was found that the cuticle containing chitin branched so as to isolate the uropod primordia and the telson primordia (Figure 2C). These findings suggest that the uropod and telson primordia are closely connected from the moment of hatching, enclosed within the cuticle, and that the separation occurs during development (Figure 2D).

Previous studies have suggested that the development of epidermal and neural tissue in some crustacean larvae is less susceptible to heterochrony than the development of mesodermal muscles.^31^^,^^32^ Our results show that the cuticle, as well as the epidermal cells, are well-developed in the early stage. This means that just prior to hatching, the patterning of the epithelium of uropod is completed. It thus appears essential to examine the patterning of epithelial tissue during the process of embryogenesis in order to gain insight into the mechanisms underlying the diversification of crustacean tail morphology. The expression of the Hox gene during embryogenesis may be related to this, as observed in the appendages of other crustaceans.^35^^,^^36^ We also found a fascinating phenomenon: cuticle branching. A comparison of this process with the molecular mechanism of tracheogenesis in insects, particularly in regard to the branching of the cuticle, could prove enlightening. It is still unclear how the epithelium derived from the uropod primordium and the epithelium derived from the telson primordium cooperate to achieve such a structure. Thus, further studies are needed in the future.

### The developmental process of primordium surface structure

**Figure 3:**
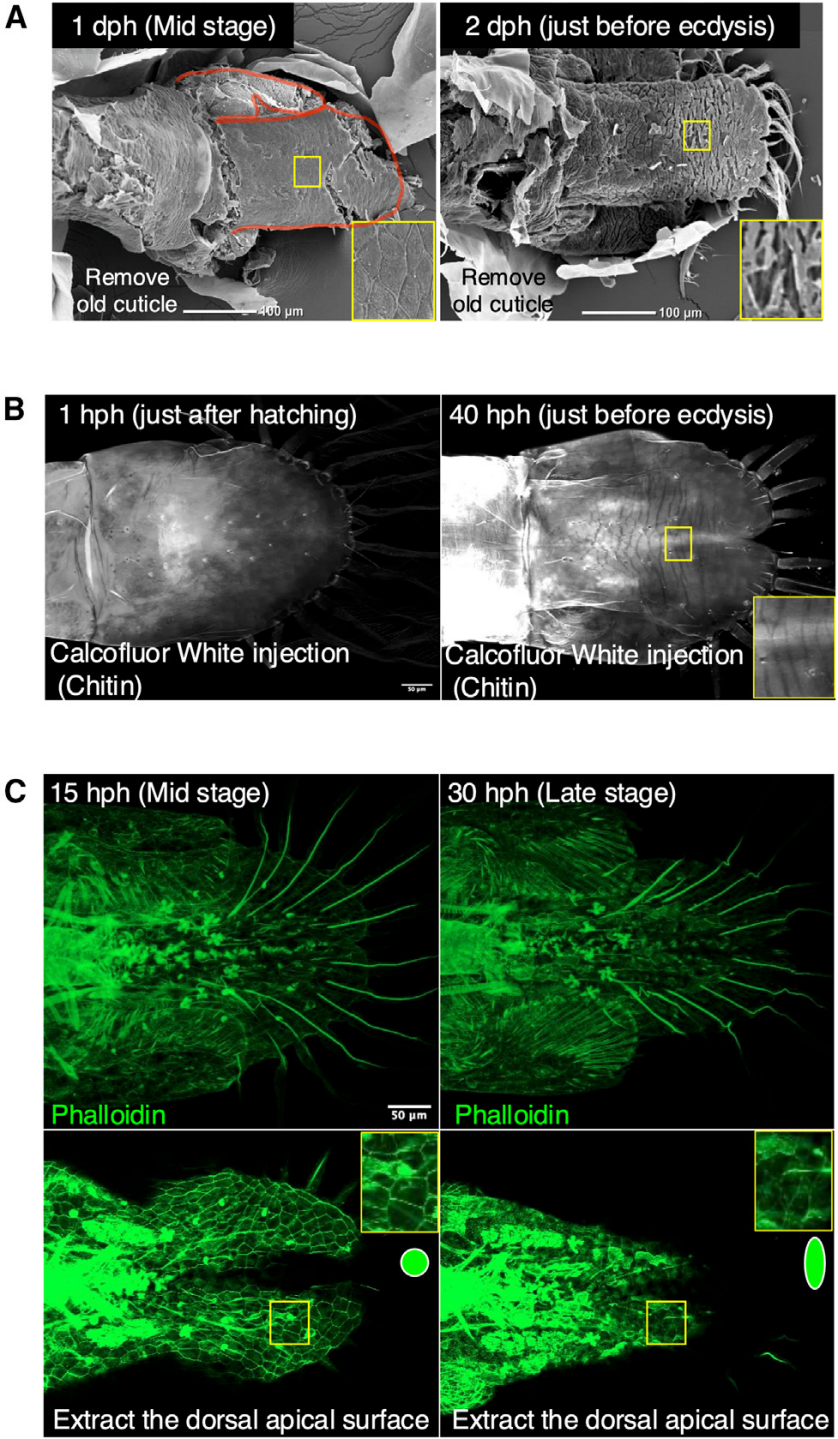
Surface structure of the developmental tail (A) Scanning electron microscopy (SEM) image of the developmental tail at 1 dph and 2 dph, taken from the dorsal side. The red outline shows the uropod and telson branches. The red arrows indicate anisotropic cellular-like shapes (left: mid-stage) and anisotropic cuticular micro furrows (right: just before ecdysis). (B) Calcofluor white injection image of developmental tail at 1hph and 40 hph. The red arrow indicates anisotropic cuticular micro furrows. (C) Phalloidin staining of developmental tail at 15 hph and 40 hph. The top image shows the complete projection, while the bottom image displays the dorsal apical planes projection. The red arrows indicate anisotropic cellular shapes.

The analysis above has enhanced our comprehension of the formation of the branching structures of the uropod and telson primordia. However, our understanding of the convergent elongation of the telson during ecdysis remains inadequate. Since the tail morphogenesis through ecdysis is a brief period of time, the existence of morphogenesis by the folding and unfolding system, as confirmed in insects,^1^ is expected. This means that the folding structure of the primordia just before ecdysis should allow such a short transformation. When the primordial surface during molting was exposed by dissection and SEM analysis was performed, anisotropic furrows were confirmed on the surface of the primordia just before ecdysis, whereas the surface of the mid-stage primordia was relatively smooth (Figure 3A). In contrast, no such structures were found on the surface after ecdysis (Figures 1A and S1). SEM analysis can produce microstructural artifacts due to the ethanol dehydration and freeze-drying processes. Therefore, the microstructure of the cuticle was also observed using calcofluor white staining with confocal microscopy. Immediately after hatching, the cuticle (chitin) of the primordia could not be identified and only the outermost cuticle could be seen. On the other hand, just before molting, the primordia could be seen in addition to the outermost cuticle (Figures 3B and S2). As in the SEM analysis, anisotropic furrow-like structures were observed in the primordia just before ecdysis (Figure 3B). These results suggested the existence of folding and extension morphogenesis in the shrimp tail similar to the morphogenesis through ecdysis in some insects.

In SEM observations, the mid-stage primordia surface was relatively smooth, but upon closer inspection, cell-like contours were discernible (Figure 3B, enlarged view). Then, phalloidin staining was performed to confirm cell-like structures detected by SEM analysis at mid-stage. The phalloidin staining method was subject to variation depending on the stage of development. Samples taken just after hatching (1 hph) and just before ecdysis (40 hph) could not be successfully stained with phalloidin for the outline of the epithelial cells, although the samples could be seen F-actin in neurons, muscles and some mesenchymal-like cells. This may be due to the cuticle, as the outline of the epithelial cells could be clearly seen when the cuticle was detached by apolysis, possibly before the formation of a new cuticle (15 hph and 30 hph; Figure 3C, Videos S3 and S4). The cell shape of the uropod primordium at 30 hph, the late stage of development, was anisotropic, corresponding to the direction of the furrows seen in SEM (Figure 3C, Video S4). On the other hand, no such anisotropy was observed in the cell shape of telson primordia at 15 h post-hatching (Figure 3C, Video S3). These results suggest that cells acquire anisotropy during development, which determines the direction of furrows. Based on these results, it appears that cuticular furrows occur intercellularly. This study suggests the existence of furrows at various scales in arthropods, including the multicellular furrows in the horns of *Trypoxylus*^1^^,^^4^ and the intracellular furrows in *Drosophila* larvae.^37^ Each is expected to have a different formation mechanism and should be studied independently and integrated them in the future.

### Establishment of *in vivo* live imaging using lectin probe

**Figure 4:**
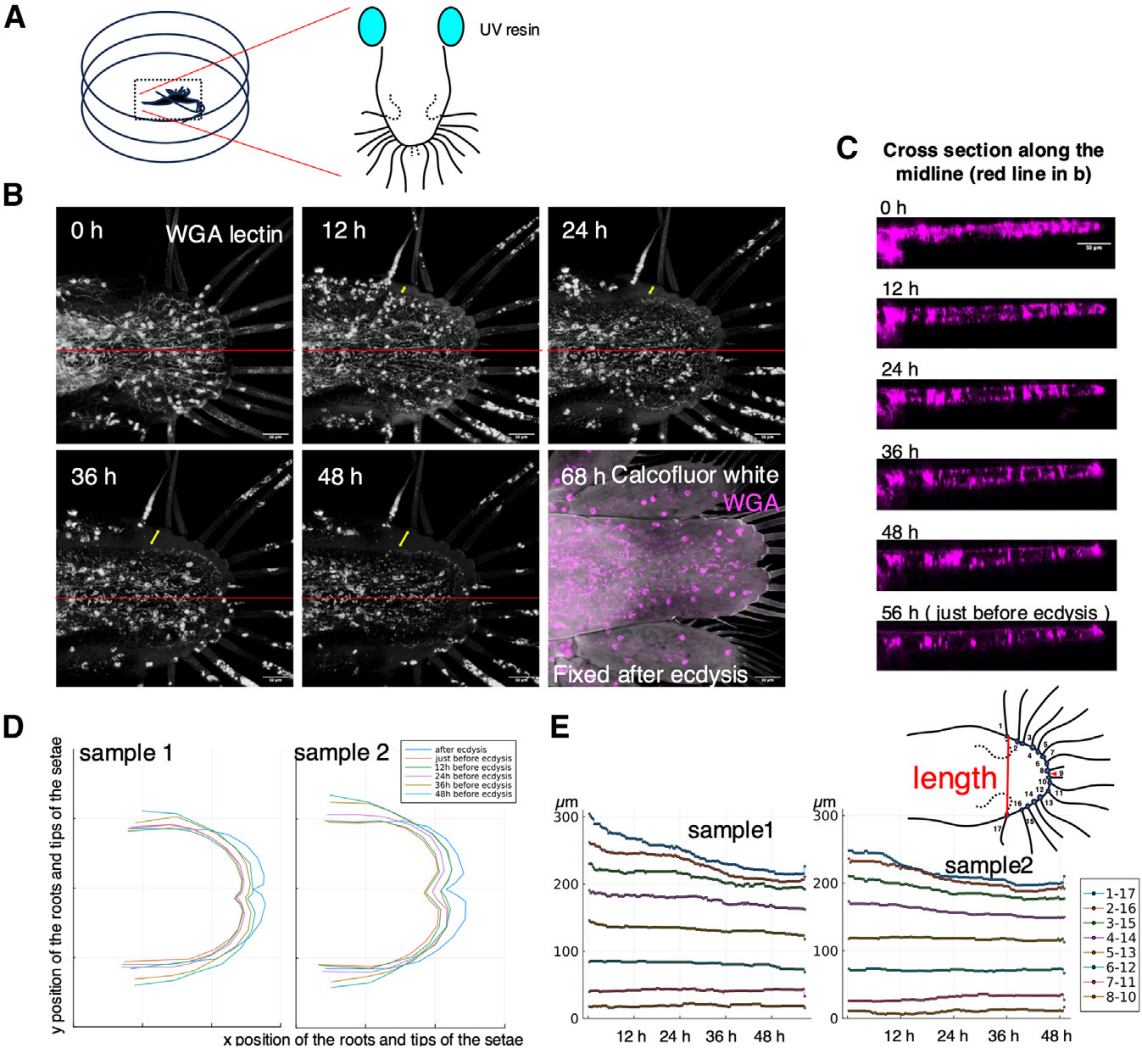
*in vivo* live imaging of the developmental process of the tail (A) Scheme of immobilization method of a living organism. The UV-LED resin was placed on either side of the caudal side of the abdomen in a glass bottom dish. (B) Time-lapse image of *in vivo* live imaging of the developmental process of the tail. Fluorescent dye-conjugated WGA-lectin injection stains vessels, neurons and hemocytes, the contours of the tissue. 68 h after starting imaging, the samples after ecdysis were fixed by 4% paraformaldehyde and stained by Calcofluor white. (C) Cross-sectional images of the time-lapse images of the red line (B). (D) The coordinates of the root and tip of the node were plotted at several time points for the two samples and connected with a straight line. (*n* = 2 animals and 17nodes). (E) Time series analysis of the width length of the developmental tail from start recording. The 8 interval lengths between the left and right setae were plotted. The final point indicates after ecdysis. (*n* = 2 animals and time interval = 20 min).

It has been proposed that the process of extension among the convergent extension is achieved through the folding and unfolding of the cuticle. Conversely, the mechanisms underlying the process of convergence among the convergent extension remain poorly understood. Accordingly, the objective was to elucidate the mechanisms of convergence through the utilization of live imaging techniques. we thought that the transparent and planar structure of the *Neocaridina* tail would facilitate live observation of the developmental process. In previous research, it was reported that an amazing *in vivo* live imaging system of *Parhyale* using surgery glue could visualize the leg regeneration process up to ecdysis.^25^ We applied this experimental system to establish an *in vivo* live imaging system using UV-LED resin (Figure 4A). The use of UV-LED resin significantly reduces the cost compared to the adhesive system used above. On the other hand, UV-LED resins generate heat during curing, so the effect of artifacts due to this must be carefully considered. In the case of *Neocaridina*, the small size of the UV-LED resin allowed them to survive and continue molting, although the time it takes from hatching to ecdysis has slightly increased. The UV-LED resin was placed on either side of the caudal side of the abdomen and the individuals were able to perform abdominal exercises. Some individuals survived ecdysis in live imaging, in which case the tail morphology was normal (Figure 4B 68 h). Our system using fluorescent dye-conjugated WGA-lectin cell labeling has allowed long-term live imaging at 20-min intervals of the organism and has been able to capture some of the cellular dynamics of the tail from the moment of hatching to the time of ecdysis (Figure 4B, Videos S5 and S6). WGA allowed the visualization of some types of cells. The tubular structures are identified as vessels, indicating that the vasculature is developed to the periphery, albeit the shrimp is in an open vasculature. A thin, fibrous structure was observed, which appeared to be a network of nerves, with some extending to the tip of the setae. It is postulated that the spherical cells which transfer rapidly are hemocytes. Additionally, the presence of slow-moving, mesenchymal-like cells was observed in areas in close proximity to the proximal gastrointestinal tract (Figures 4B and S3). Moreover, the time-lapse images were capable of capturing some of the tissue contours and demonstrating the contraction of the entire tissue (Figure 4B yellow two direction arrow). Observation of the time series of midline optical cross-sections showed that the thickness of the tissue itself did not change much (Figure 4C). Fluctuations in pressure caused by the movement of blood cells have the potential to result in alterations to tissue thickness. Nevertheless, the lack of a notable alteration in thickness could suggests the potential existence of a mechanism that maintains tissue thickness. For instance, dorsal-ventral intercellular projections in the *Drosophila* wing could serve as possible linkages (39), and the dynamics have been investigated (40). The presence of such structures could be crucial for maintaining thickness. Also, the formation of epidermal folding (Figure 2) in such an environment with fluctuations in pressure remains a significant enigma that necessitates further investigation.

Video S5. *in vivo* live imaging of the tail development_sample1 CF555 conjugated WGA was injected into ∼3 hph larva.

Video S6. *in vivo* live imaging of the tail development_sample2 Alexa Fluor 647 conjugated WGA was injected into around 10 hph larva.

There are 16 setae on the telson of *Neocaridina*. The morphological changes in the telson were analyzed based on the coordinates of the roots of the setae and tips of the telson (Figure 4D, Videos S7 and S8). In agreement with the results of the fixed tissue analysis (Figure 1D), it was observed that the width of the telson narrows depend on the development, particularly in the proximal site (Figure 4E). After ecdysis, the interval was shorter at many locations compared to before ecdysis, but longer at the most proximal locations (Figure 4E). This difference may be attributed to the directionality of microscopic furrows at the most proximal sites (Figure 3A). It was found that hemocytes circulate even during the contraction process of the telson (Videos S5 and S6), suggesting the presence of contractile force generation beyond pressure due to circulation of hemocytes. The mechanism by which contraction occurs is not clear at present, but the TUNEL assay did not detect a wide range of apoptosis in the tail developmental process (Figure S4). The potential for future live imaging to elucidate the role of apoptosis in morphogenesis is a possibility, but the prevailing view at this stage is that the processes of cell contraction and rearrangement are more significant in influencing tissue contraction. Tissue shrinkage occurs in various species and scales. Research has shown that morphogenesis in *Drosophila* imaginal discs and beetle horns is influenced by contraction and adhesion.^38^^,^^39^ Additionally, convergent extension occurs in the embryonic development of various animal species, demonstrating two different mechanistic models of collective cell movements.^40^ In the future, it is important to consider comparisons with the molecular mechanisms of such phenomena.

Video S7. Morphological changes in the telson_sample1 Manual tracking of the coordinates of the roots and tips of the setae.

Video S8. Morphological changes in the telson_sample2 Manual tracking of the coordinates of the roots and tips of the setae.

During the course of live observations, we noticed that before ecdysis there were hemocyte like cells between the old cuticle and the epithelial tissue. These cells frequently extended their pseudopodia and moved actively during development. They also swelled, burst and died during ecdysis (Video S5, Figure S5). It is thought that ecdysis allowed fresh water to enter from the outside, and the osmotic pressure caused the cells to rupture. Some fluorescence staining images also showed cells outside the epithelial tissue. Apolysis is known to occur in the molting process of conventional arthropods and the gaps are filled with molting fluid, but we are not aware of any reported cases where cells are present. Previous studies have mainly used fixed tissues for analysis, so they may have been washed away in the process. However, in the *Parhyale* leg regeneration experiment, motile cells, probably phagocytes, were reported as “data not shown” in the space between the exoskeleton and the epidermis.^25^ It is possible that there was an injury as artifact of live imaging in our experiment. However, at least the space between the old cuticle and the epithelial tissue was found to be a viable environment for the cells. Additionally, the previous cuticle, which has shed its epithelium through apolysis and seems to be decomposing, may possess a sturdy framework that inhibits freshwater from penetrating the interior until just before ecdysis.

The success of prolonged *in vivo* live imaging of the tail during molt, in addition to enabling the dynamics of convergence along the convergent elongation to be traced, has prompted the formulation of a number of new research questions.

### Whole genome sequence for expansion of genetic tool

The above analysis shows the developmental process of the tail of *Neocaridina*. This also indicates that *Neocaridina* can be used for *in vivo* live imaging of cell dynamics. However, this study only labeled certain cell types. To gain further insight into morphogenesis, it is essential to monitor the behavior of shaped epithelial cells and their surrounding cells and extracellular matrix over time. To monitor the dynamics of a specific cell type and the other proteins, fluorescent transgenic individuals are often generated in model organisms. However, it should be noted that *Neocaridina* currently has only short-read sequence information or unpublished genome assemblies.^41^^,^^42^^,^^43^ To address this issue, we present a long-read genome sequence of *N. denticulata* in this study. By utilizing only the nanopore sequence, we were able to capture a draft genome of 2.73 Gb with 36,889 contigs. The longest sequence length was 933 kb. The N50 sequence length was 123 kb and the GC content was 35%. The BUSCO analysis revealed that approximately 80% of the genes in the arthropods database were complete (Table 1). This sequence can be utilized in the future for various genetic tools, such as fluorescent transgenic lines and detailed live imaging.

**Table 1:** Statistics of the genome assembly

**Length Statistics and Composition**	

Number of sequences:	36,889
Total length (nt):	2,728,854,793
Longest sequence (nt):	933,025
N50 sequence length (nt):	122,794
GC-content (%)	34.69

**BUSCO Score (arthropoda_odb10)**	

Complete (%)	80.85
Complete + Partial (%)	91.41

### Limitations of the study

In the *in vivo* live imaging performed in this study, individuals were fixed using UV-LED resin, but UV-LED resin generates heat during fixation, so the possibility of artifacts due to this effect must be considered. Furthermore, the live imaging technique employed in this study was unable to visualize all the cells present in the sample. It is therefore crucial to develop genetic labeling techniques based on the draft genomic data obtained in this study. For example, there are a number of gene labeling methods that could be employed, including the identification of promoters to generate transgenic individuals or knock-in experiments using CRISPR/Cas9. However, this is a task that will be addressed in the future.

The molecular mechanisms for the branching formation of the uropods and the phenomenon of convergent elongation of the telson are not at all clear in the present study; in *Neocaridina*, in principle, knockdown and knockout experiments are possible,^33^^,^^44^ so further work is needed to elucidate the molecular mechanisms using these techniques.

It is also important to highlight the universality of the phenomena that have been revealed in this study. The larval styles of crustaceans vary depending on the species. The subject of this study, *N. denticulata*, is a direct developmental form with a morphology almost identical to that of the adult, except for the tail.^29^^,^^30^ In other shrimp species that undergo zoea larvae, a divergent structure appears in subsequent stages, similar to the present content. However, it is currently unclear whether the phenomenon of convergent elongation of the telson is present in other species. A comparative analysis of some of the phenomena identified in the present study in other species will have to be carried out in due course.

The study has not also been able to ascertain the subjects’ sex, which might indicate the presence of sex disparities.

### Conclusion

Analysis of morphological changes in the tail showed that two major phenomena occur before and after the first ecdysis in *N. denticulata*. One is a clear branching of the uropod and telson, and the other is a convergent extension of the telson. Cross-sectional analysis revealed that uropod and telson branching occurs immediately after hatching in the form of cuticle branching. The surface structure of the developmental tail suggests that telson elongation is achieved by the extension of anisotropic furrows in the cuticle during ecdysis. Anisotropy of cuticle furrows is associated with cell shape, and anisotropy of cell shape is found to occur during development from post-hatching. *in vivo* live imaging analysis shows that telson contraction occurs more proximal and gradually prior to ecdysis. These results also suggest that *Neocaridina* is useful for cellular and developmental analysis in crustaceans. This study also generated a draft genome of *Neocaridina*, which may stimulate future cell and developmental biology studies with this species.

## Resource availability

### Lead contact

Further information and requests for resources and reagents should be directed to and will be fulfilled by the Lead Contact, Haruhiko Adachi (hrhk.adachi@gmail.com).

### Materials availability

This study did not generate new unique reagents.

### Data and code availability

• The draft genome data and the original sequencing data have been deposited at DDBJ under BioProject accession number PRJDB17690.
• This study did not report original code.
• Any additional information required to reanalyze the data reported in this paper is available from the lead contact upon request.

## Acknowledgments

We appreciate Prof. Shigeru Kondo (Osaka University) and Prof. Shizue Ohsawa (Nagoya University) and Prof. Teruyuki Niimi (National Institute for Basic Biology) and Prof. Noriko Funayama (Kyoto University) and their laboratory members for providing an environment for research and helpful discussion. We thank Prof. Seiji Takashima (Shinshu University) and Prof. Hiroki Gotoh (Shizuoka University) for providing some reagents and helpful discussion and Dr. Yusuke Fuke (National Institute of Genetics) for helpful discussion about species identification. We also thank G-language group (Institute for Advanced Biosciences) for helpful supports and discussion. This research was supported in part by MEXT KAKENHI Grant Number 23K14115 (to H.A.), 22J00193 (to H.A.) and 22KJ1542 (to H.A.) and 10.13039/501100011907Mishima Kaiun Memorial Foundation (to H.A.) and 10.13039/100020108Fuji Seal Foundation (to H.A.) and research funds from the Yamagata Prefectural Government and Tsuruoka City, Japan. H.A. was also supported by Grant-in-Aid for JSPS Fellows (P.D.).

## Author contributions

Conceptualization: H.A.; investigation: H.A.; formal analysis: H.A.; funding acquisition: H.A. and K.A.; methodology: H.A.; resources: H.A. and K.A.; TEM analysis: N.M. and T.S.; setting up genome sequence systems: K.A.; writing—original draft: H.A.; writing—review and editing: all authors.

## Declaration of interests

The authors have no competing interests.

## Declaration of generative AI and AI-assisted technologies in the writing process

During the preparation of this work the authors used DeepL in order to enhance the clarity and improve the grammatical usage of English. After using this tool/service, the authors reviewed and edited the content as needed and takes full responsibility for the content of the publication.

## STAR★Methods

### Key resources table

**Table:** 

REAGENT or RESOURCE	SOURCE	IDENTIFIER
**Chemicals, peptides, and recombinant proteins**		

Clove oil	Fujifilm Wako	8000-34-8
Alexa Fluor 488 Phalloidin	Thermo Fisher Scientific	A12379
Hoechst 33342	Nacalai Tesque	23491-52-3
SYTO13	Thermo Fisher Scientific	S7575
Calcofluor White	MP Biomedicals	4404-43-7
CF555 WGA	Biotium	29076-1
Alexa Fluor 647 WGA	Thermo Fisher Scientific	W32466
UV-LED resin	Padico	403242
CF640R TUNEL Assay Apoptosis Detection Kit	Biotium	30074

**Software and algorithms**		

ImageJ	Schneider et al.^45^	https://imagej.nih.gov/ij/
Flye	Pavel Pevzner's lab at UCSD^46^	https://github.com/mikolmogorov/Flye
Guppy	Oxford Nanopore Technologies	https://nanoporetech.com/ja/document/Guppy-protocol
Raven	Vaser^47^	https://github.com/lbcb-sci/raven
Quickmerge	Chakraborty et al.^48^	https://github.com/mahulchak/quickmerge
ntLink	Coombe et al.^49^^,^^50^	https://github.com/bcgsc/ntLink
Purge_haplotigs	Roach et al.^51^	https://bitbucket.org/mroachawri/purge_haplotigs/src/master/
CAT	Meijenfeldt et al.^52^	https://github.com/MGXlab/CAT_pack
gVolante	Nishimura et al.^53^	https://gvolante.riken.jp/

**Deposited data**		

Draft genome	This paper	Accession number: PRJDB17690 https://www.ncbi.nlm.nih.gov/nuccore/BAABUL000000000

**Other**		

Smart Coater	JEOL	DII-29010SCTR
Scanning Electron Microscope	JEOL	JCM-6000
Freeze-dried system	Asahi Life Science	FZ-2.5
Confocal Microscope	Carl Zeiss	LSM900
Fluorescence Microscope	Keyence	BZ-X 710
Transmission Electron Microscope	JEOL	JEM-1400plus
Ultramicrotome	Leica	UC7
Automated electrophoresis system	Agilent	TapeStation 2200
Sequence platform	Oxford Nanopore Technologies	PromethION
Microinjector	BEX	BJ-120
Puller	Narishige	PC-100

### Experimental model and study participant details

#### Animals

*Neocaridina denticulata* (*Neocaridina davidi*) were commercially purchased and kept in 45 L water tanks at 23-25°C. The tanks were checked daily to identify individuals carrying eggs, which were then transferred to separate bottles. Day 0 was defined as the first day the eggs hatched. Individuals used in time series analysis were checked for hatching every three hours. The hatched individuals were transferred to 96-well plates along with 300 μL of water. The 1st stage larvae mostly underwent ecdysis 40-50 hours after hatching. Movies of the molting were recorded with a BZ-X710 (Keyence, Japan). The captured individuals molted faster, likely due to the higher temperature inside the microscope. The experiments were performed after anesthesia with clove oil diluted approximately 10,000 times. Species identification was performed using partial CO1 sequences based on recommendation of Dr. Yusuke Fuke (National Institute of Genetics, Japan) (Figure S6).^54^ The genus *Neocaridina* includes many closely related species, leading to ongoing debate about the correct species name. Recent study proposed that the genus *Neocaridina* should be classified as a single species, *Neocaridina denticulata*, based on the genetic distance observed in the mitochondrial genome.^55^ Given the ongoing nature of the debate, this paper employs the use of *Neocaridina denticulata* (*Neocaridina davidi*) as its subject.

### Method details

#### Scanning electron microscopy (SEM)

The samples were fixed with 4% paraformaldehyde (PFA) in PBS (-) for 24 hours at 4°C and dehydrated using a series of ethanol (50-100%). After dehydration, they were soaked in t-butanol and dried using a freeze-dry system (FZ-2.5; Asahi Life Science, Japan). For observation of the tail primordium, the old cuticle was removed by dissection, using tweezers. Finally, Au sputtering was conducted on the samples with DII-29010SCTR Smart Coater (JEOL Ltd., Japan) and they were observed using a scanning electron microscope JCM-6000plus (JEOL, Japan) with 10 kV. The study was conducted on three individuals at each developmental stage.

#### Transmission electron microscopy (TEM)

The tails of the samples were isolated and fixed in a solution of 2% paraformaldehyde and 2% glutaraldehyde in PBS (-) at 4°C overnight, before being replaced with PBS. The samples were fixed in 1% osmium tetroxide/0.1 M phosphate buffer at 4°C for 2 hours. They were then dehydrated using a series of ethanol (50-100%) and replaced with QY-1. The samples were then replaced with resin and embedded before being polymerized. The samples were sectioned using an ultramicrotome UC7 (Leica, Germany). Semi-sections cut to around 1 μm were stained by 0.5% toluidine blue. The samples were observed by optical microscope. Ultrathin sections, cut to a thickness of 70-80 nm, were stained with Uranyl acetate solution for 15 minutes, followed by Pb staining solution for 10 minutes. The samples were observed by Transmission electron microscope JEM-1400plus (JEOL, Japan) with 100 kV. The study was conducted on two individuals at each developmental stage.

#### Fluorescence staining

The tails of the samples were isolated and fixed in 4% PFA 4°C. The fixed samples were treated with 0.1% Triton-X 100 in PBS (-) for 10 min and the samples were soaked in staining solution with 0.1 % Tween-20 in PBS (-). SYTO13 (Thermo Fisher Scientific, USA) was used at a dilution concentration of 1:1000, and Alexa Fluor 488 conjugated phalloidin (Cell Signaling Technology, USA) was used at a dilution concentration of 1:100. Calcofluor White (MP Biomedicals, USA) and CF®555 conjugated WGA (Biotium, USA) or Alexa Fluor 647 Conjugated WGA (Thermo Fisher Scientific, USA) were injected into the living body using microinjector BJ-120 (BEX, Japan) and glass capillary needle. Calcofluor White was used at a mass concentration of 5%, and WGA was used at a concentration of 1 mg/ml. Glass capillary needles were prepared by puller PC-100 (Narishige, Japan). The injection time was set to 'short' mode, and the solution was injected 2-3 times into the larva's abdomen. For live imaging, the injected individuals were immobilized in the center of a glass bottom dish (Matsunami, Japan) using UV-LED resin (Padico, Japan) and 30 seconds of 395 nm light irradiation. Images were captured using confocal laser scanning microscopy LSM 900 with Axio Observer.Z1 (Zeiss, Germany). Plan-Apochromat 20x/0.8 M27 and Plan-Apochromat 10x/0.45 M27, and GaAsP PMT detector were used. For live imaging, time-lapse images were captured at 20-minute intervals and z-stack imaging was performed at 2μm intervals. The images were processed with ImageJ-Fiji version 2.14.0.^45^ Projection figure generated by standard deviation projection type. The TUNEL assay was performed using the CF(R)640R TUNEL Assay Apoptosis Detection Kit (Biotium, USA) with fixed tissue according to the kit manual. Each study was conducted on three or more individuals.

#### Analysis of the morphology of the telson

Images were analyzed with ImageJ-Fiji version 2.14.0.^45^ The length of the telson was measured from the root of the uropod to the tip of the telson. The widths of the telson were measured between the second setae on the proximal side. coordinates of the roots and tips of the setae. For the analysis of the time-lapse images, the coordinates of the roots and tips of the setae were obtained using the "Manual Tracking" plugin. Coordinate information was processed by the Julia language version 1.8. The 8-interval lengths between the left and right setae were calculated from the coordinate information. The study was conducted on two individuals.

#### Genome sequence and genome assembly

Genomic-tip 20/G (QIAGEN, Netherlands) was used to extract genomic DNA from three adult individuals according to the manufacturer's protocol. The eluted DNA was resuspended in 10 mM Tris-HCl, quantified using Qubit Broad Range (BR) dsDNA assay (Life Technologies, USA) and qualified using TapeStation 2200 with Genomic DNA Screen Tape (Agilent Technologies, USA). Sequencing was performed on a P2 Solo instrument (Oxford Nanopore Technologies, UK) using a PromethION R10.4.1 flow cell. The data obtained were base called using Guppy version 6.4.6 with the 'super accurate' base calling mode, and the adapters were trimmed. Reads from each of the three sequencing runs underwent filtration to remove those less than 1000 bp each. Combined set of reads also underwent filtration to remove those less than 4000 bp. Four sets of sequences were prepared due to the computer's memory limitations. Flye version 2.9.1 was used to assemble sequenced reads from each of the three sequencing runs, as well as from a combined set of reads. The combined set of reads was also assembled by raven-assembler version 1.8.3.^46^^,^^47^ The five assemblies were combined into a single assembly using quickmerge version 0.3.^48^ Subsequently, the assembly was scaffolded three times using ntlink version 1.3.9 and Flye polishing version 2.9.1.^49^^,^^50^ Haplotype duplication was removed and the mitochondrial genome was eliminated using purge_haplotigs version 1.1.2.^51^ Contamination was also identified and removed using CAT version 5.3.^52^ The draft genome was validated using BUSCO v.5^56^ with the core arthropods (Arthropoda_odb10 database) on the gVolante webserver.^53^ The sequences were uploaded to DDBJ under BioProject accession number PRJDB17690.

### Quantification and statistical analysis

The number of experimental repeats is indicated for each experiment or corresponding figure legend.
