# Supplementary material for: Post-embryonic tail development through molting of the freshwater shrimp Neocaridina denticulata
Source: iScience. 2025 Jan 23;28(2):111885. doi: 10.1016/j.isci.2025.111885 (PMC11883442; doi:10.1016/j.isci.2025.111885)
Supplement: Document S1. Figures S1–S6 [file mmc1.pdf]

iScience, Volume *28*

Supplemental information

Post-embryonic tail development through molting of the freshwater shrimp *Neocaridina denticulata*

Haruhiko Adachi, Nobuko Moritoki, Tomoko Shindo, and Kazuharu Arakawa

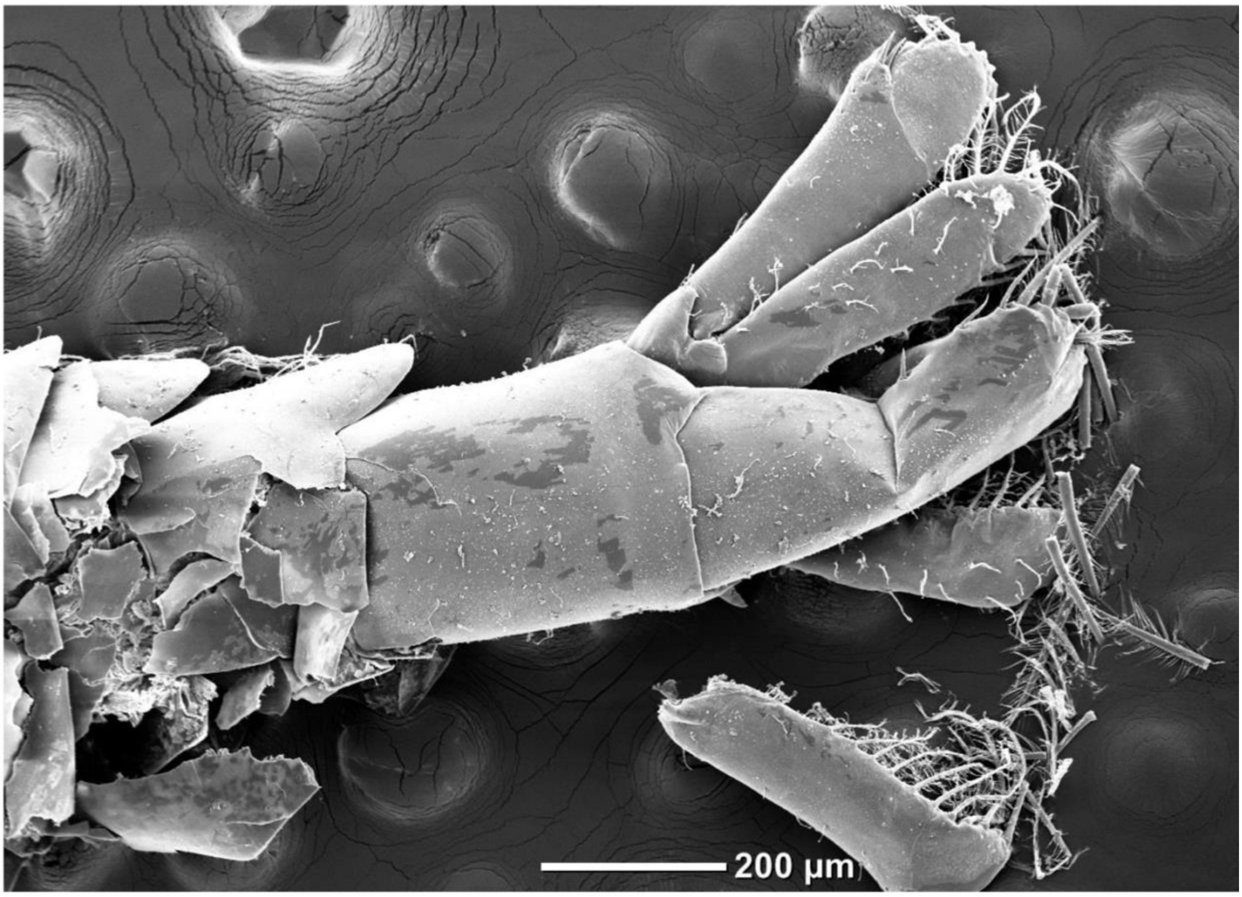

Fig. S1 Surface structure of the tail in the 2nd instar

SEM image of the tail of *Neocaridina*, 2nd instar (2 days post-hatch), taken from the dorsal side. A detailed examination revealed the absence of any discernible micro furrows on the surface. It was observed that some tissue had been torn off during the dissection process.

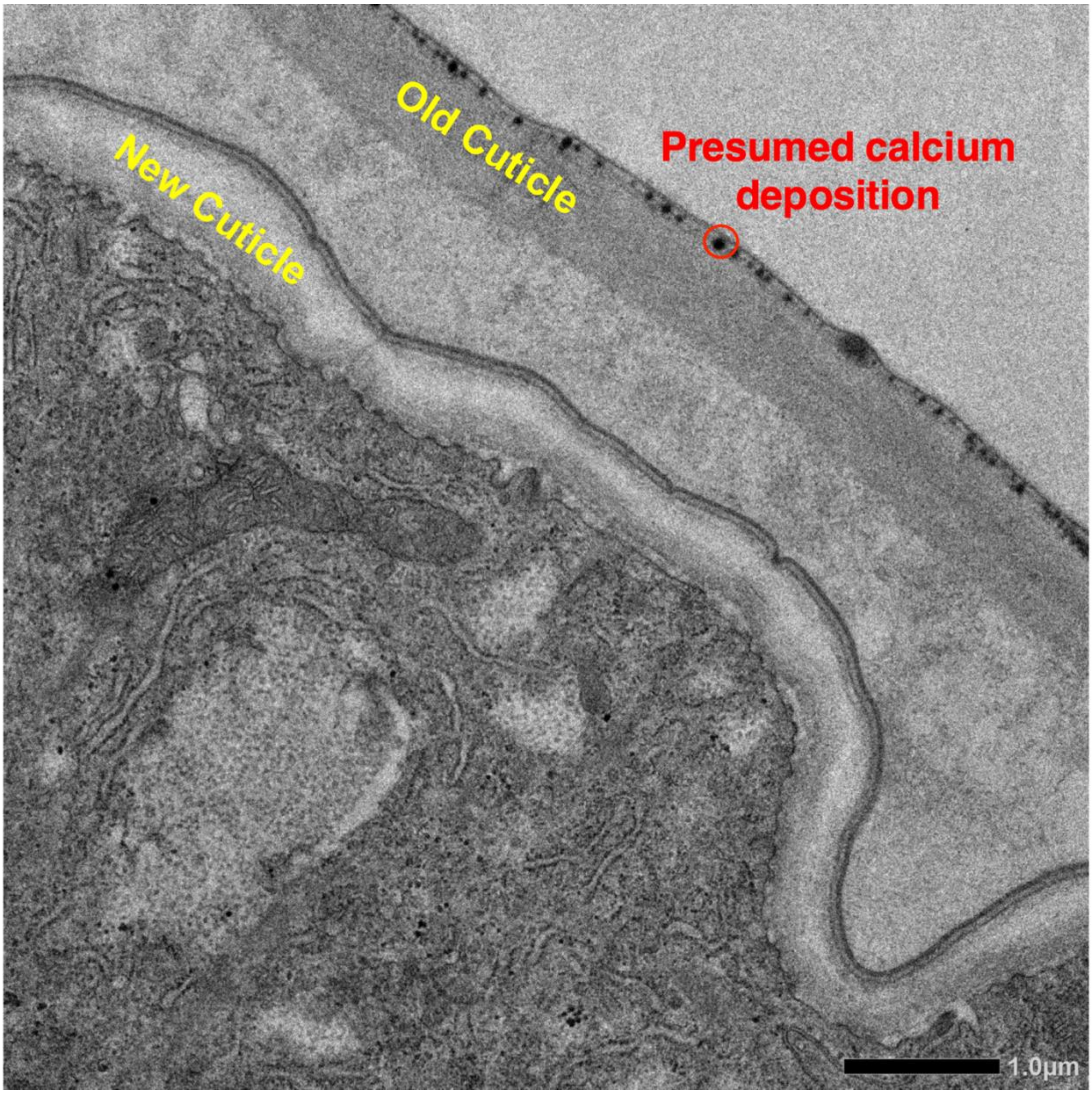

Fig. S2 Transverse sectional structure of the tail just before ecdysis

Transmission electron microscopy (TEM) image of the tail in 40-50 hph. In addition to the old cuticle, a new cuticle of the primordia can also be identified. The old cuticle displays the presence of structures that appear to be calcium deposits, whereas the new cuticle lacks such structures.

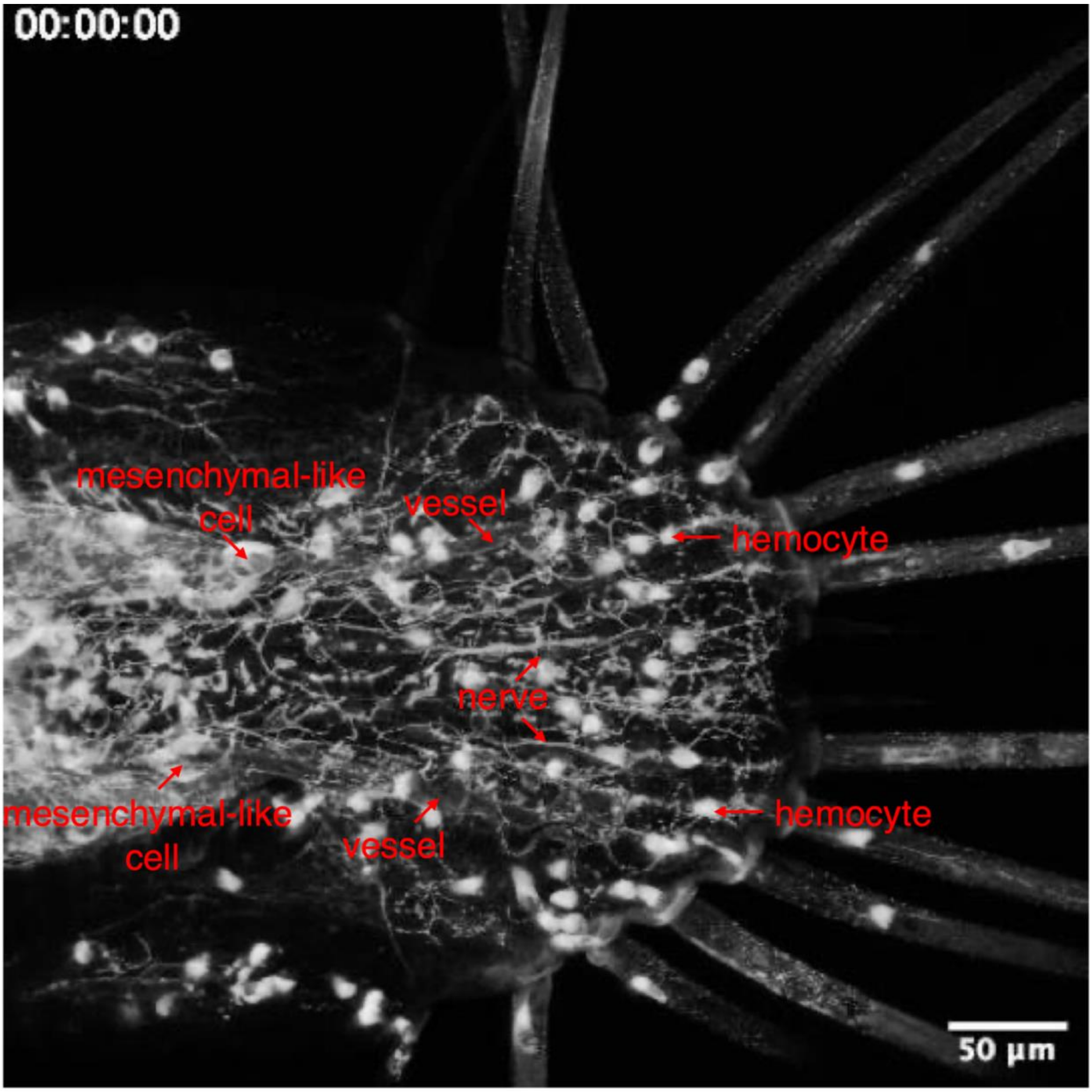

Fig. S3 Description of the differences between each cell in live imaging

Video clips (initial images) of live imaging with injected fluorescently labelled WGA illustrate the differences between the stained cells. Based on cell outline and movement, the cells were classified as haemocyte, mesenchymal-like cell, nerve and vessel.

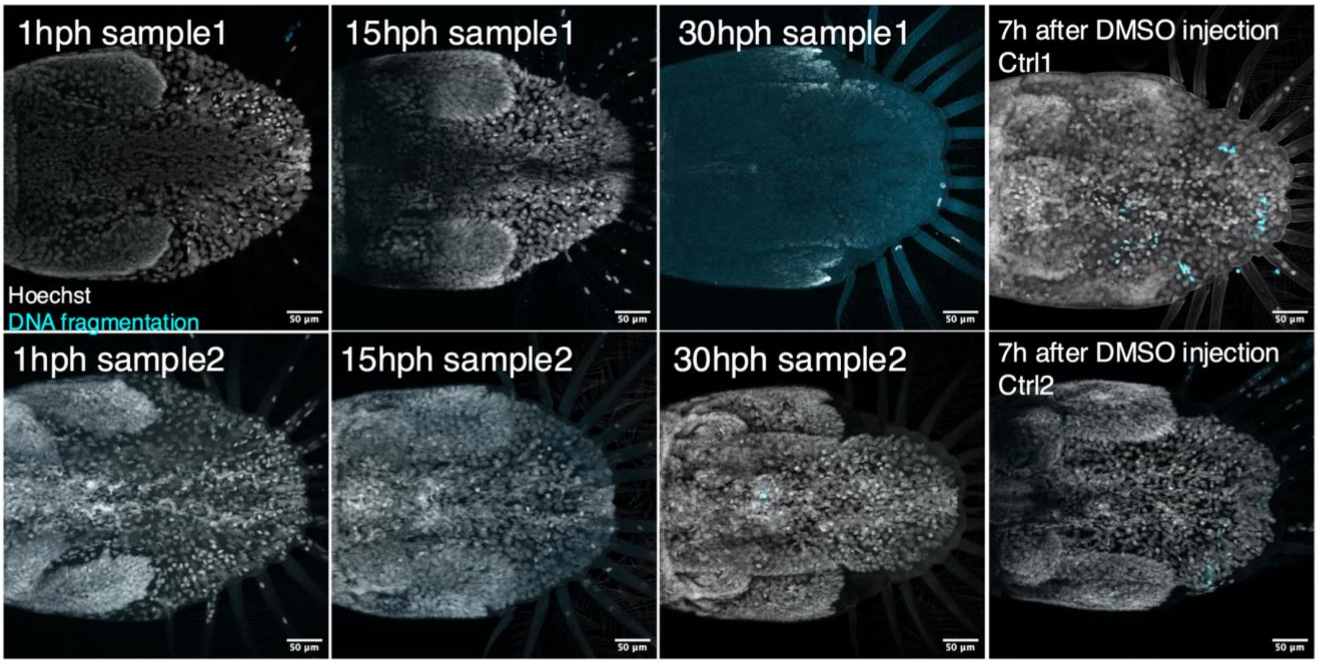

Fig. S4 TUNEL assay during the tail development process

Detection of apoptosis during the tail development process. The absence of TUNEL-positive cell nuclei overlapping with the Hoechst-stained image in the tails at 1, 15, and 30 hours post-hatching was confirmed. In the tails prepared as positive controls and injected with DMSO after hatching and 7 hours post-injection, the presence of TUNEL-positive cell nuclei was identified, with these nuclei overlapping with the Hoechst-stained image.

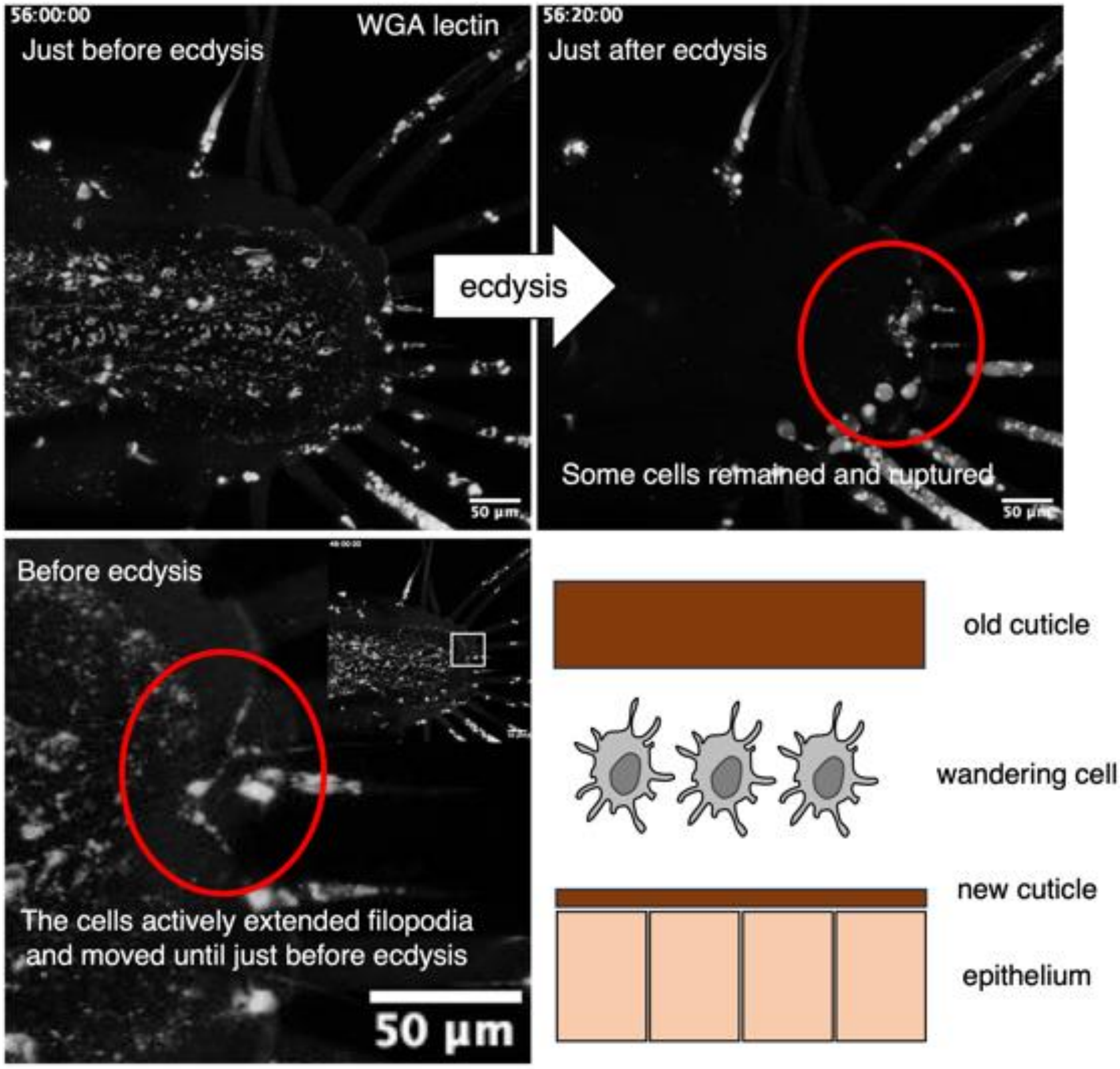

Fig. S5 The presence of cells between the old cuticle and epithelial tissue

Time-lapse images just before and after ecdysis. Some cells remained and ruptured after ecdysis, and the cells actively extended filipodia and moved until just before ecdysis.

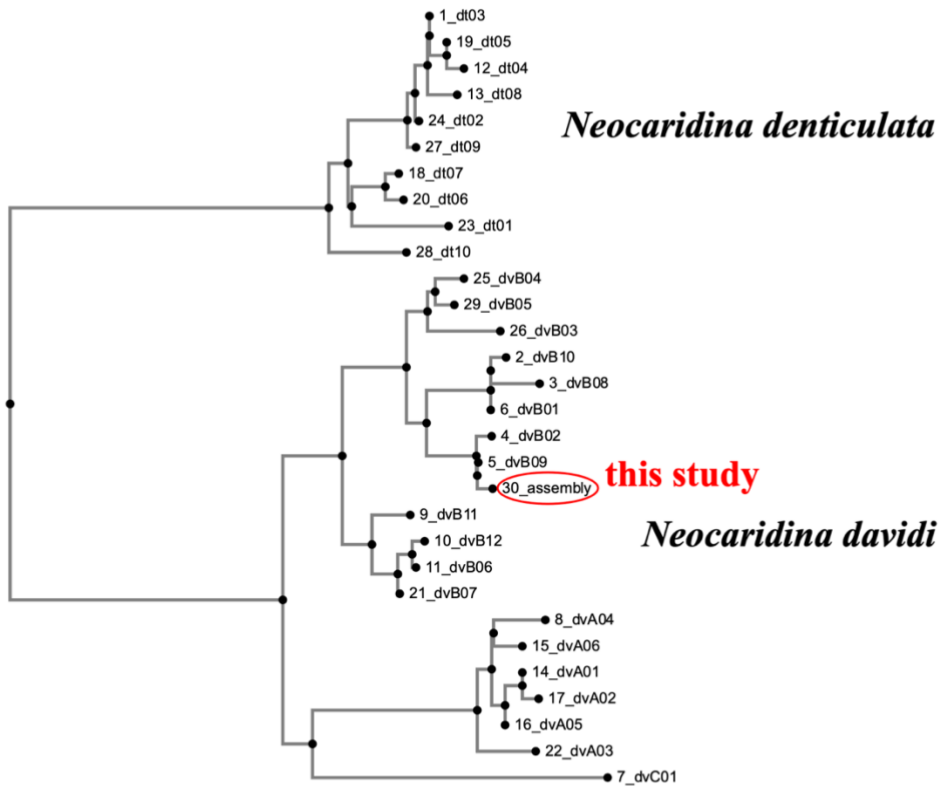

Fig. S6 Phylogenetic trees for species identification

Phylogenetic tree using Partial CO1 sequences established in previous studies (45). The phylogenetic tree was constructed using the Neighbour-Joining method.
